# Intermittent, Remittent, and Congestive Fevers

**Published:** 1845-02

**Authors:** 


// THE WESTERN JOURNAL.

New Series.—Vol. III.—No. II.

LOUISVILLE, FEBRUARY 1, 1845.

### INTERMITTENT, REMITTENT, AND CONGESTIVE FEVER.

Dr. Barbour, Professor of Obstetrics in Kemper College, has lately published, at the request of his class, an excellent pamphlet on the above subjects. He gives his own observations on these diseases, and the practice which he lays down is that in which a large majority of the physicians of our country would unite in recommending. He is an advocate for the liberal use of ice and iced drinks, and for small doses of calomel, and large doses of quinine—the practice which is beginning to obtain in all the regions where the purging system once prevailed so exclusively. In fevers of the remittent type his experience is, that quinine given during the remission, is as efficacious as it is in intermittent fever. We like his suggestion of *muriatic acid* as a refrigerant in fevers. He directs a drachm of the acid, diluted with a pint of iced or cold spring water, to be drank in the course of the day. He thinks it acts favorably on the liver, being often sufficient of itself, after the use of mild aperients, to correct the secretions of that organ. In chronic intermittents—cases in which, on slight exposure, chills continue to return, for a long time—we have found the preparations of iron more effectual than any other remedies. Change of air, especially from miasmatic to healthy regions, is doubtless beneficial, and the alterative medicines mentioned by Dr. B. are indicated in those cases which are complicated with visceral enlargements; but in a majority of instances patients affected with this form of the disease exhibit the signs of anemia, and are cured by remedies which improve the condition of the blood. Nothing does this so effectually as iron in its various states. Y.

